# *trans*-(2-Benzoyl­pyridine-κ^2^ *N*,*O*)dichlorido[2-(2-pyridylcarbon­yl)phenyl-κ^2^ *C* ^1^,*N*]iridium(III) dichloro­methane solvate

**DOI:** 10.1107/S1600536809004322

**Published:** 2009-02-13

**Authors:** Mao-Lin Hsueh, Cheng-Hsien Yang

**Affiliations:** a Nano-Powder and Thin Film Technology Center, ITRI South, Tainan 709, Taiwan

## Abstract

The title compound, [Ir(C_12_H_8_NO)Cl_2_(C_12_H_9_NO)]·CH_2_Cl_2_, which was obtained from the reaction of iridium(III) chloride trihydrate and 2-benzoyl­pyridine, contains an Ir^III^ atom coordinated by two N, one O, one C and two Cl atoms in *trans* positions, forming a distorted octa­hedral environment. The solvent molecule CH_2_Cl_2_ is disordered over two positions with an occupancy of 0.8:0.2.

## Related literature

For the synthesis and structure of Rh(Hbzpy)(bzpy)Cl_2_ (bzpy is 2-pyridyl-2-phenonide), see: de Geest & Steel (1995 ▶). For a related structure, see: Tseng *et al.* (2005 ▶).
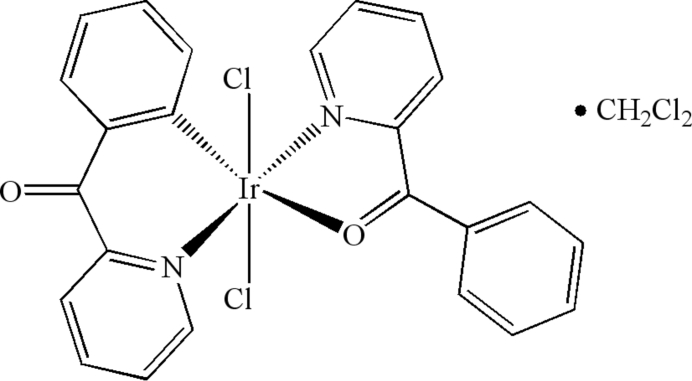


## Experimental

### 

#### Crystal data

[Ir(C_12_H_8_NO)Cl_2_(C_12_H_9_NO)]·CH_2_Cl_2_
*M* *_r_* = 713.42
Triclinic, 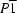

*a* = 8.8694 (9) Å
*b* = 11.4600 (11) Å
*c* = 13.2604 (13) Å
α = 113.543 (2)°
β = 95.719 (2)°
γ = 90.641 (2)°
*V* = 1227.6 (2) Å^3^
*Z* = 2
Mo *K*α radiation
μ = 5.90 mm^−1^
*T* = 294 (2) K
0.13 × 0.13 × 0.08 mm

#### Data collection

Bruker SMART 1000 CCD area-detector diffractometer
Absorption correction: multi-scan (*SADABS*; Sheldrick, 1996 ▶) *T*_min_ = 0.480, *T*_max_ = 0.624
14570 measured reflections
6075 independent reflections
5416 reflections with *I* > 2σ(*I*)
*R*_int_ = 0.032

#### Refinement

*R*[*F* ^2^ > 2σ(*F* ^2^)] = 0.020
*wR*(*F* ^2^) = 0.044
*S* = 0.85
6075 reflections
325 parameters
H-atom parameters constrained
Δρ_max_ = 0.65 e Å^−3^
Δρ_min_ = −0.52 e Å^−3^

### 

Data collection: *SMART* (Bruker, 2007 ▶); cell refinement: *SAINT* (Bruker, 2007 ▶); data reduction: *SAINT*; program(s) used to solve structure: *SHELXS97* (Sheldrick, 2008 ▶); program(s) used to refine structure: *SHELXL97* (Sheldrick, 2008 ▶); molecular graphics: *SHELXTL* (Sheldrick, 2008 ▶); software used to prepare material for publication: *SHELXTL*.

## Supplementary Material

Crystal structure: contains datablocks global, I. DOI: 10.1107/S1600536809004322/fi2071sup1.cif

Structure factors: contains datablocks I. DOI: 10.1107/S1600536809004322/fi2071Isup2.hkl

Additional supplementary materials:  crystallographic information; 3D view; checkCIF report

## Figures and Tables

**Table:** 

Ir1—C24	1.995 (2)
Ir1—N2	2.032 (2)
Ir1—N1	2.047 (2)
Ir1—O1	2.1983 (17)
Ir1—Cl2	2.3407 (8)
Ir1—Cl1	2.3416 (7)

**Table:** 

C24—Ir1—N2	88.46 (9)
C24—Ir1—N1	99.89 (9)
N2—Ir1—N1	171.15 (7)
C24—Ir1—O1	174.73 (8)
N2—Ir1—O1	95.21 (7)
N1—Ir1—O1	76.67 (7)
C24—Ir1—Cl2	91.26 (7)
N2—Ir1—Cl2	92.19 (6)
N1—Ir1—Cl2	90.59 (6)
O1—Ir1—Cl2	84.84 (6)
C24—Ir1—Cl1	91.73 (7)
N2—Ir1—Cl1	88.61 (6)
N1—Ir1—Cl1	88.19 (6)
O1—Ir1—Cl1	92.14 (6)
Cl2—Ir1—Cl1	176.93 (2)

**Fig. 1.**
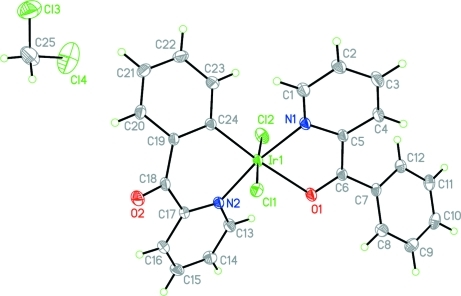
A view of the molecular structure of the title compound with displacement ellipsoids shown at the 50% probability level.

## supplementary crystallographic information

### Comment

Several studies have been reported in cyclometallation of orthometallated ligands such as anionic ppy (ppyH is 2–phenylpyridine). For instance, Rh(Hbzpy)(bzpy)Cl_2_ (bzpy is 2–pyridyl–2–phenonide) was obtained from the reaction of rhodium (III) chloride trihydrate with 2–benzoylpyridine (Hbzpy) in 2–methoxyethanol for 4 d at room temperature (de Geest & Steel, 1995). The orthometallated complex which they reported had a five–membered *N*,*O* metallacycle containing the chelated Hbzpy ligand and a six–membered *N*,*C* metallacycle containing the chelated bzpy ligand, on the basis of NMR chemical–shift analysis. Moveover, the structural study of the orthometallated Rh^III^ complex was reported in order to make sure the stereochemistry (Tseng *et al.*, 2005). However, the synthesis and structural study of the according iridium complex was never reported. We report herein the synthesis and characterization studies of the title orthometallated Ir^III^ complex, (I), containing 2–pyridyl phenone.

In the orthometallated title compound, the Ir atom is hexacoordinated by two equivalents of 2–benzoylpyridine, forming a pseudo–octahedral geometry, with two Cl ligands in a *trans* orientation. The pyridyl N atom of the Hbzpy ligand is *trans* to the N atom of the bzpy ligand, where one of the ligands is κ^2^–(*N*,*C*)–cyclometallated and the other is κ^2^–(N,*O*)–coordinated. Cyclometallation leads to a boat conformation, with atoms Ir and C18 above the N2—C17—C19—C24 plane (Fig. 1). The pyridyl ring of the Hbzpy ligand and the phenyl ring of the bzpy ligand are mutually stacked [C1—N1—C24—C23 = 48.8 (2)°] (Table 1). The five-membered chelate ring deviates slightly from planarity [N1—C5—C6—O1 = -10.9 (3)°] and is inclined to the phenyl plane [C5—C6—C7—C12 = -39.1 (4)°]. There are no short intermolecular contacts.

### Experimental

All procedures involving Ir(III) species were carried out under nitrogen gas atomosphere. 2-Benzoylpyridine (10.0 mmol) and 0.4 equiv. of IrCl_3_.H_2_O (Next Chimica) were heated in a 3:1 mixture of 2-ethoxyethanol and water. This slurry was heated to 100 °C for 24 hours. After cooling to room temperature, the precipitate was filtered off and washed with deionized water, followed by 2 portions of *n*-hexane and ether. The orange–reddish single crystals were obtained from the solutions of dichlomathane and *n*-hexane mixture (1:1) in 43% yield.

### Refinement

All H atoms bonded to C atoms were placed in calculated positions, with C—H = 0.96 Å, and treated as riding atoms, with *U*_iso_(H) = 1.2*U*_eq_(C). The solvent molecule CH_2_Cl_2 _was refined as disordered with an 80/20 occupancy for the two molecules.

### Crystal data

**Table:**

[Ir(C_12_H_8_NO)Cl_2_(C_12_H_9_NO)]·CH_2_Cl_2_	*Z* = 2
*M**_r_* = 713.42	*F*(000) = 688
Triclinic, *P*1	*D*_x_ = 1.930 Mg m^−^^3^
Hall symbol: -P 1	Mo *K*α radiation, λ = 0.71073 Å
*a* = 8.8694 (9) Å	Cell parameters from 8499 reflections
*b* = 11.4600 (11) Å	θ = 2.3–28.3°
*c* = 13.2604 (13) Å	µ = 5.90 mm^−^^1^
α = 113.543 (2)°	*T* = 294 K
β = 95.719 (2)°	Equant, orange–red
γ = 90.641 (2)°	0.13 × 0.13 × 0.08 mm
*V* = 1227.6 (2) Å^3^	

### Data collection

**Table:**

Bruker SMART 1000 CCD area-detector diffractometer	6075 independent reflections
Radiation source: fine-focus sealed tube	5416 reflections with *I* > 2σ(*I*)
graphite	*R*_int_ = 0.032
φ and ω scans	θ_max_ = 28.3°, θ_min_ = 1.7°
Absorption correction: multi-scan (*SADABS*; Sheldrick, 1996)	*h* = −11→11
*T*_min_ = 0.480, *T*_max_ = 0.624	*k* = −15→15
14570 measured reflections	*l* = −17→17

### Refinement

**Table:**

Refinement on *F*^2^	Primary atom site location: structure-invariant direct methods
Least-squares matrix: full	Secondary atom site location: difference Fourier map
*R*[*F*^2^ > 2σ(*F*^2^)] = 0.020	Hydrogen site location: inferred from neighbouring sites
*wR*(*F*^2^) = 0.044	H-atom parameters constrained
*S* = 0.85	*w* = 1/[σ^2^(*F*_o_^2^) + (0.025*P*)^2^] where *P* = (*F*_o_^2^ + 2*F*_c_^2^)/3
6075 reflections	(Δ/σ)_max_ = 0.003
325 parameters	Δρ_max_ = 0.65 e Å^−^^3^
0 restraints	Δρ_min_ = −0.52 e Å^−^^3^

### Special details

**Table:**

Geometry. All esds (except the esd in the dihedral angle between two l.s. planes) are estimated using the full covariance matrix. The cell esds are taken into account individually in the estimation of esds in distances, angles and torsion angles; correlations between esds in cell parameters are only used when they are defined by crystal symmetry. An approximate (isotropic) treatment of cell esds is used for estimating esds involving l.s. planes.
Refinement. Refinement of *F*^2^ against ALL reflections. The weighted *R*-factor *wR* and goodness of fit *S* are based on *F*^2^, conventional *R*-factors *R* are based on *F*, with *F* set to zero for negative *F*^2^. The threshold expression of *F*^2^ > σ(*F*^2^) is used only for calculating *R*-factors(gt) *etc*. and is not relevant to the choice of reflections for refinement. *R*-factors based on *F*^2^ are statistically about twice as large as those based on *F*, and *R*-factors based on ALL data will be even larger.

### Fractional atomic coordinates and isotropic or equivalent isotropic displacement parameters (Å^2^)

**Table:**

	*x*	*y*	*z*	*U*_iso_*/*U*_eq_	Occ. (<1)
Ir1	0.792178 (11)	0.696938 (8)	0.230068 (7)	0.03493 (4)	
Cl1	1.05493 (7)	0.69691 (6)	0.22304 (5)	0.04395 (14)	
Cl2	0.53142 (8)	0.69236 (7)	0.24425 (7)	0.05771 (18)	
Cl3	0.3748 (3)	1.1051 (3)	0.34837 (18)	0.1197 (8)	0.80
Cl3'	0.4493 (10)	1.0756 (13)	0.3508 (9)	0.146 (5)	0.20
Cl4	0.5216 (4)	1.0079 (3)	0.1454 (3)	0.1488 (12)	0.80
Cl4'	0.5595 (8)	1.0593 (7)	0.1793 (8)	0.0678 (17)	0.20
O1	0.8137 (2)	0.65126 (17)	0.37670 (14)	0.0475 (4)	
O2	1.0387 (2)	0.98256 (19)	0.15186 (16)	0.0568 (5)	
N1	0.7869 (2)	0.50192 (19)	0.16390 (16)	0.0380 (5)	
N2	0.8146 (2)	0.88896 (19)	0.31697 (16)	0.0363 (4)	
C1	0.8068 (3)	0.4270 (3)	0.0594 (2)	0.0477 (6)	
H1A	0.8174	0.4645	0.0097	0.057*	
C2	0.8123 (4)	0.2965 (3)	0.0224 (2)	0.0530 (7)	
H2A	0.8241	0.2471	−0.0513	0.064*	
C3	0.8004 (4)	0.2402 (3)	0.0952 (2)	0.0555 (7)	
H3A	0.8033	0.1523	0.0717	0.067*	
C4	0.7840 (3)	0.3160 (2)	0.2038 (2)	0.0459 (6)	
H4A	0.7778	0.2797	0.2548	0.055*	
C5	0.7767 (3)	0.4459 (2)	0.23675 (19)	0.0366 (5)	
C6	0.7796 (3)	0.5377 (2)	0.3539 (2)	0.0378 (5)	
C7	0.7543 (3)	0.4988 (2)	0.4445 (2)	0.0373 (5)	
C8	0.8372 (3)	0.5651 (3)	0.5475 (2)	0.0481 (6)	
H8A	0.9069	0.6308	0.5568	0.058*	
C9	0.8169 (3)	0.5345 (3)	0.6353 (2)	0.0537 (7)	
H9A	0.8751	0.5775	0.7031	0.064*	
C10	0.7108 (3)	0.4405 (3)	0.6233 (2)	0.0551 (7)	
H10A	0.6969	0.4202	0.6831	0.066*	
C11	0.6252 (3)	0.3764 (3)	0.5236 (3)	0.0533 (7)	
H11A	0.5517	0.3143	0.5167	0.064*	
C12	0.6468 (3)	0.4032 (3)	0.4328 (2)	0.0450 (6)	
H12A	0.5902	0.3579	0.3648	0.054*	
C13	0.7676 (3)	0.9380 (3)	0.4180 (2)	0.0471 (6)	
H13A	0.7084	0.8859	0.4390	0.057*	
C14	0.8026 (3)	1.0616 (3)	0.4918 (2)	0.0537 (7)	
H14A	0.7660	1.0928	0.5606	0.064*	
C15	0.8931 (3)	1.1386 (3)	0.4622 (2)	0.0518 (7)	
H15A	0.9221	1.2217	0.5116	0.062*	
C16	0.9394 (3)	1.0901 (2)	0.3583 (2)	0.0451 (6)	
H16A	0.9994	1.1409	0.3364	0.054*	
C17	0.8971 (3)	0.9661 (2)	0.28598 (19)	0.0360 (5)	
C18	0.9372 (3)	0.9224 (2)	0.1688 (2)	0.0387 (5)	
C19	0.8380 (3)	0.8238 (2)	0.07782 (19)	0.0347 (5)	
C20	0.8219 (3)	0.8370 (3)	−0.0232 (2)	0.0449 (6)	
H20A	0.8799	0.8999	−0.0317	0.054*	
C21	0.7218 (4)	0.7582 (3)	−0.1096 (2)	0.0538 (7)	
H21A	0.7106	0.7677	−0.1763	0.065*	
C22	0.6371 (3)	0.6638 (3)	−0.0963 (2)	0.0558 (7)	
H22A	0.5685	0.6099	−0.1546	0.067*	
C23	0.6534 (3)	0.6491 (3)	0.0025 (2)	0.0465 (6)	
H23A	0.5944	0.5860	0.0099	0.056*	
C24	0.7564 (3)	0.7267 (2)	0.09154 (19)	0.0353 (5)	
C25	0.4019 (5)	0.9802 (4)	0.2225 (4)	0.1002 (15)	
H25A	0.3050	0.9535	0.1787	0.120*	
H25B	0.4373	0.9095	0.2379	0.120*	

### Atomic displacement parameters (Å^2^)

**Table:**

	*U*^11^	*U*^22^	*U*^33^	*U*^12^	*U*^13^	*U*^23^
Ir1	0.04842 (6)	0.02687 (5)	0.03220 (5)	−0.00222 (4)	0.01063 (4)	0.01344 (4)
Cl1	0.0475 (3)	0.0455 (4)	0.0425 (3)	0.0045 (3)	0.0056 (3)	0.0214 (3)
Cl2	0.0544 (4)	0.0489 (4)	0.0736 (5)	−0.0046 (3)	0.0270 (4)	0.0242 (4)
Cl3	0.1304 (19)	0.152 (2)	0.0675 (10)	−0.0255 (16)	0.0092 (13)	0.0358 (11)
Cl3'	0.109 (7)	0.167 (8)	0.096 (5)	0.037 (6)	−0.024 (5)	−0.007 (5)
Cl4	0.149 (3)	0.167 (3)	0.169 (3)	0.087 (2)	0.083 (2)	0.091 (3)
Cl4'	0.044 (2)	0.061 (3)	0.109 (5)	0.009 (2)	0.018 (2)	0.041 (3)
O1	0.0776 (13)	0.0303 (9)	0.0373 (9)	−0.0051 (9)	0.0133 (9)	0.0151 (7)
O2	0.0651 (12)	0.0562 (13)	0.0538 (11)	−0.0219 (10)	0.0086 (10)	0.0273 (10)
N1	0.0512 (12)	0.0299 (11)	0.0350 (10)	−0.0028 (9)	0.0117 (9)	0.0137 (8)
N2	0.0469 (12)	0.0308 (11)	0.0324 (10)	0.0003 (9)	0.0086 (9)	0.0130 (8)
C1	0.0682 (18)	0.0369 (15)	0.0391 (14)	0.0024 (13)	0.0185 (13)	0.0138 (11)
C2	0.075 (2)	0.0367 (15)	0.0416 (15)	0.0030 (14)	0.0187 (14)	0.0066 (12)
C3	0.080 (2)	0.0307 (14)	0.0547 (17)	0.0071 (14)	0.0191 (15)	0.0129 (12)
C4	0.0611 (17)	0.0328 (14)	0.0483 (15)	0.0044 (12)	0.0143 (13)	0.0191 (11)
C5	0.0454 (13)	0.0298 (12)	0.0375 (12)	−0.0006 (10)	0.0092 (10)	0.0158 (10)
C6	0.0463 (14)	0.0319 (13)	0.0381 (13)	0.0002 (10)	0.0101 (11)	0.0162 (10)
C7	0.0463 (14)	0.0343 (13)	0.0386 (13)	0.0057 (10)	0.0126 (11)	0.0206 (10)
C8	0.0560 (17)	0.0466 (16)	0.0422 (14)	−0.0025 (13)	0.0112 (12)	0.0172 (12)
C9	0.0562 (17)	0.067 (2)	0.0419 (15)	0.0082 (15)	0.0104 (13)	0.0252 (14)
C10	0.0626 (18)	0.069 (2)	0.0555 (17)	0.0187 (15)	0.0243 (14)	0.0431 (16)
C11	0.0570 (17)	0.0520 (18)	0.0675 (19)	0.0052 (14)	0.0209 (15)	0.0382 (15)
C12	0.0485 (15)	0.0426 (15)	0.0497 (15)	−0.0014 (12)	0.0089 (12)	0.0240 (12)
C13	0.0620 (17)	0.0388 (15)	0.0414 (14)	0.0015 (12)	0.0155 (12)	0.0150 (11)
C14	0.0711 (19)	0.0451 (17)	0.0376 (14)	0.0057 (14)	0.0140 (13)	0.0074 (12)
C15	0.0624 (18)	0.0326 (14)	0.0482 (16)	0.0008 (13)	−0.0004 (13)	0.0050 (12)
C16	0.0512 (15)	0.0324 (14)	0.0494 (15)	−0.0036 (11)	0.0021 (12)	0.0151 (11)
C17	0.0417 (13)	0.0304 (12)	0.0381 (12)	0.0000 (10)	0.0025 (10)	0.0166 (10)
C18	0.0445 (14)	0.0340 (13)	0.0442 (13)	−0.0014 (10)	0.0060 (11)	0.0225 (11)
C19	0.0396 (13)	0.0317 (12)	0.0363 (12)	0.0013 (10)	0.0060 (10)	0.0170 (10)
C20	0.0576 (16)	0.0434 (15)	0.0423 (14)	0.0062 (12)	0.0117 (12)	0.0248 (12)
C21	0.072 (2)	0.0552 (18)	0.0359 (14)	0.0118 (15)	0.0021 (13)	0.0203 (13)
C22	0.0597 (18)	0.0560 (18)	0.0416 (15)	0.0008 (14)	−0.0089 (13)	0.0125 (13)
C23	0.0472 (15)	0.0422 (15)	0.0481 (15)	−0.0083 (12)	−0.0005 (12)	0.0176 (12)
C24	0.0386 (12)	0.0334 (13)	0.0350 (12)	0.0005 (10)	0.0074 (10)	0.0142 (10)
C25	0.087 (3)	0.069 (3)	0.137 (4)	−0.010 (2)	−0.016 (3)	0.040 (3)

### Geometric parameters (Å, °)

**Table:**

Ir1—C24	1.995 (2)	C8—H8A	0.9300
Ir1—N2	2.032 (2)	C9—C10	1.372 (4)
Ir1—N1	2.047 (2)	C9—H9A	0.9300
Ir1—O1	2.1983 (17)	C10—C11	1.370 (4)
Ir1—Cl2	2.3407 (8)	C10—H10A	0.9300
Ir1—Cl1	2.3416 (7)	C11—C12	1.386 (4)
Cl3—C25	1.753 (5)	C11—H11A	0.9300
Cl3'—C25	1.621 (12)	C12—H12A	0.9300
Cl3'—Cl4'	2.504 (17)	C13—C14	1.372 (4)
Cl4—C25	1.656 (5)	C13—H13A	0.9300
Cl4'—C25	1.911 (9)	C14—C15	1.379 (4)
O1—C6	1.239 (3)	C14—H14A	0.9300
O2—C18	1.220 (3)	C15—C16	1.372 (4)
N1—C1	1.338 (3)	C15—H15A	0.9300
N1—C5	1.366 (3)	C16—C17	1.381 (3)
N2—C17	1.349 (3)	C16—H16A	0.9300
N2—C13	1.342 (3)	C17—C18	1.511 (3)
C1—C2	1.379 (4)	C18—C19	1.478 (3)
C1—H1A	0.9300	C19—C24	1.402 (3)
C2—C3	1.370 (4)	C19—C20	1.401 (3)
C2—H2A	0.9300	C20—C21	1.367 (4)
C3—C4	1.376 (4)	C20—H20A	0.9300
C3—H3A	0.9300	C21—C22	1.388 (4)
C4—C5	1.377 (3)	C21—H21A	0.9300
C4—H4A	0.9300	C22—C23	1.380 (4)
C5—C6	1.485 (3)	C22—H22A	0.9300
C6—C7	1.473 (3)	C23—C24	1.395 (3)
C7—C8	1.394 (3)	C23—H23A	0.9300
C7—C12	1.395 (3)	C25—H25A	0.9600
C8—C9	1.370 (4)	C25—H25B	0.9600
			
C24—Ir1—N2	88.46 (9)	C12—C11—H11A	119.7
C24—Ir1—N1	99.89 (9)	C7—C12—C11	119.4 (3)
N2—Ir1—N1	171.15 (7)	C7—C12—H12A	120.3
C24—Ir1—O1	174.73 (8)	C11—C12—H12A	120.3
N2—Ir1—O1	95.21 (7)	N2—C13—C14	123.1 (3)
N1—Ir1—O1	76.67 (7)	N2—C13—H13A	118.5
C24—Ir1—Cl2	91.26 (7)	C14—C13—H13A	118.5
N2—Ir1—Cl2	92.19 (6)	C13—C14—C15	118.8 (3)
N1—Ir1—Cl2	90.59 (6)	C13—C14—H14A	120.6
O1—Ir1—Cl2	84.84 (6)	C15—C14—H14A	120.6
C24—Ir1—Cl1	91.73 (7)	C16—C15—C14	118.6 (2)
N2—Ir1—Cl1	88.61 (6)	C16—C15—H15A	120.7
N1—Ir1—Cl1	88.19 (6)	C14—C15—H15A	120.7
O1—Ir1—Cl1	92.14 (6)	C15—C16—C17	120.2 (3)
Cl2—Ir1—Cl1	176.93 (2)	C15—C16—H16A	119.9
C25—Cl3'—Cl4'	49.7 (4)	C17—C16—H16A	119.9
C25—Cl4'—Cl3'	40.4 (4)	N2—C17—C16	121.1 (2)
C6—O1—Ir1	112.63 (15)	N2—C17—C18	121.0 (2)
C1—N1—C5	117.9 (2)	C16—C17—C18	117.8 (2)
C1—N1—Ir1	125.79 (17)	O2—C18—C19	122.3 (2)
C5—N1—Ir1	115.96 (16)	O2—C18—C17	117.7 (2)
C17—N2—C13	118.2 (2)	C19—C18—C17	119.3 (2)
C17—N2—Ir1	122.56 (17)	C24—C19—C20	120.9 (2)
C13—N2—Ir1	118.29 (17)	C24—C19—C18	123.0 (2)
N1—C1—C2	122.7 (2)	C20—C19—C18	115.9 (2)
N1—C1—H1A	118.7	C21—C20—C19	120.6 (3)
C2—C1—H1A	118.7	C21—C20—H20A	119.7
C1—C2—C3	119.3 (3)	C19—C20—H20A	119.7
C1—C2—H2A	120.4	C20—C21—C22	119.1 (3)
C3—C2—H2A	120.4	C20—C21—H21A	120.5
C4—C3—C2	118.9 (3)	C22—C21—H21A	120.5
C4—C3—H3A	120.6	C23—C22—C21	120.7 (2)
C2—C3—H3A	120.6	C23—C22—H22A	119.6
C5—C4—C3	119.8 (2)	C21—C22—H22A	119.6
C5—C4—H4A	120.1	C22—C23—C24	121.5 (2)
C3—C4—H4A	120.1	C22—C23—H23A	119.2
N1—C5—C4	121.3 (2)	C24—C23—H23A	119.2
N1—C5—C6	114.1 (2)	C23—C24—C19	117.0 (2)
C4—C5—C6	124.1 (2)	C23—C24—Ir1	121.60 (18)
O1—C6—C7	118.8 (2)	C19—C24—Ir1	121.33 (17)
O1—C6—C5	117.9 (2)	Cl4—C25—Cl3'	108.8 (5)
C7—C6—C5	123.2 (2)	Cl4—C25—Cl3	117.7 (3)
C8—C7—C12	118.9 (2)	Cl3'—C25—Cl3	25.3 (3)
C8—C7—C6	118.1 (2)	Cl4—C25—Cl4'	19.2 (3)
C12—C7—C6	123.0 (2)	Cl3'—C25—Cl4'	89.9 (6)
C9—C8—C7	120.7 (3)	Cl3—C25—Cl4'	99.2 (3)
C9—C8—H8A	119.7	Cl4—C25—H25A	107.8
C7—C8—H8A	119.7	Cl3'—C25—H25A	132.0
C10—C9—C8	120.0 (3)	Cl3—C25—H25A	108.1
C10—C9—H9A	120.0	Cl4'—C25—H25A	121.6
C8—C9—H9A	120.0	Cl4—C25—H25B	107.2
C11—C10—C9	120.3 (3)	Cl3'—C25—H25B	90.2
C11—C10—H10A	119.9	Cl3—C25—H25B	108.2
C9—C10—H10A	119.9	Cl4'—C25—H25B	111.5
C10—C11—C12	120.7 (3)	H25A—C25—H25B	107.4
C10—C11—H11A	119.7		
